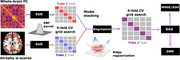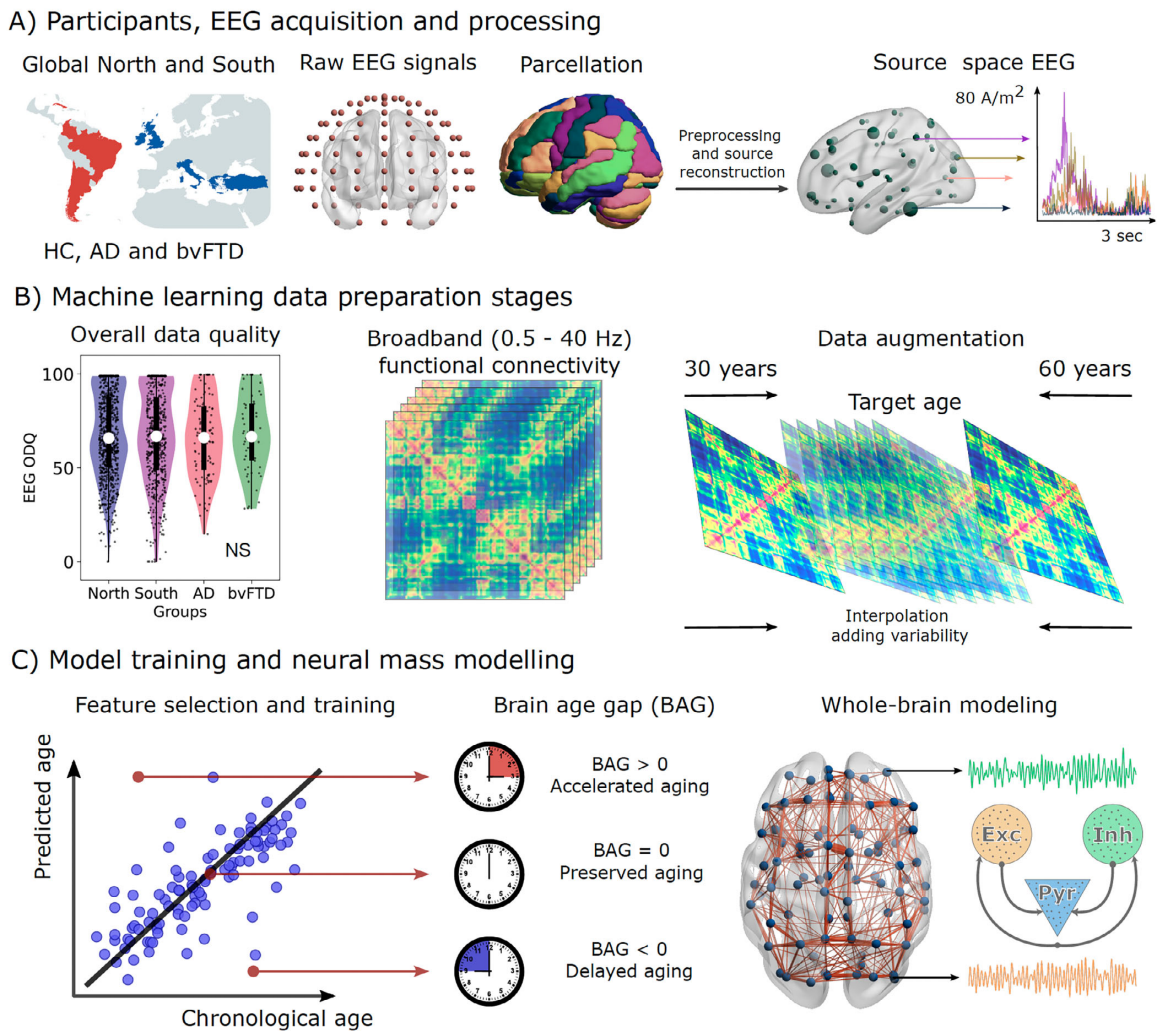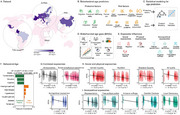# From Biophysical Mechanisms of Brain Clocks to Biobehavioral Age Gaps in Global Settings

**DOI:** 10.1002/alz70860_100264

**Published:** 2025-12-23

**Authors:** Agustin Ibanez

**Affiliations:** ^1^ BrainLat Institute, Santiago, Santiago, Chile; Global Brain Health Institute (GBHI), University of California San Francisco (UCSF); & Trinity College Dublin, Dublin, Leinster, Ireland

## Abstract

**Background:**

Advancing brain health research requires transitioning from biophysical models to broader, more generalizable frameworks that integrate environmental and socioeconomic factors. This structured synthesis highlights findings from three studies: two employing biophysical modeling with EEG and fMRI and a third introducing biobehavioral clocks to address global health disparities.

**Study 1: Biophysical Modeling with EEG**

This study investigated brain age gaps (BAGs) in aging and dementia across diverse populations (*N* = 1,399), including Alzheimer's disease (AD) and frontotemporal dementia patients. By combining EEG source‐space connectivity and generative modeling, it identified mechanisms of hyperexcitability and structural disintegration in healthy aging and hypoexcitability in dementia. BAGs were influenced by geography (South > North), income (low > high), and education levels, revealing significant disparities in accelerated aging.

**Study 2: Biophysical Modeling with fMRI**

Using multimodal structural and functional MRI data from 1,084 healthy adults, this study developed a support vector machine regressor to predict brain age. Findings highlighted decreased inter‐areal coupling and increased neural inhibition in patients with accelerated brain aging. The model effectively contrasted regional disparities in healthy brain aging, particularly between Latin America and the USA, offering mechanistic insights into how socioeconomic inequalities shape neural aging.

**Study 3: Biobehavioral Clocks in Global Contexts**

Expanding beyond biophysical mechanisms, this study analyzed protective and risk factors in 161,981 participants from 40 countries. BAGs were calculated using machine learning, integrating data on cognition, cardiometabolic health, and exposome disparities. Protective factors (e.g., education, preserved cognition) were linked to delayed aging, while risk factors (e.g., sensory impairments, gender inequality) accelerated it. Cross‐regional analyses revealed faster aging in low‐income regions, particularly Africa and Latin America, compared to Europe and Asia. High BAGs predicted future functional and cognitive declines, emphasizing their translational utility.

**Conclusion:**

This synthesis bridges biophysical and biobehavioral models, demonstrating a transition from mechanistic precision to global applicability. While biophysical models reveal neural mechanisms, biobehavioral clocks integrate these insights with environmental and socioeconomic contexts to address disparities, inform interventions, and shape policies for brain health globally.